# Dural arteriovenous fistulas and headache features: an observational study

**DOI:** 10.1186/s10194-020-1073-1

**Published:** 2020-01-16

**Authors:** Ilenia Corbelli, Francesca De Maria, Paolo Eusebi, Michele Romoli, Gabriela Cardaioli, Mohammed Hamam, Piero Floridi, Letizia Maria Cupini, Paola Sarchielli, Paolo Calabresi

**Affiliations:** 1 Clinica Neurologica, Dipartimento di Medicina, Ospedale S.M. Misericordia, Università degli Studi di Perugia, Misericordia - S. Andrea delle Fratte, 06156 Perugia, Italy; 2 Servizio di Angiografia Interventistica, Ospedale S.M. Misericordia, Perugia, Italy; 3 Servizio di Neuroradiologia, Ospedale S.M. Misericordia, Università degli Studi di Perugia, Perugia, Italy; 4 Centro Cefalee e Malattie Cerebrovascolari, UOC Neurologia-Stroke Unit, Ospedale S. Eugenio, Rome, Italy; 5 Clinica Neurologica, Dipartimento di Neuroscienze, Policlinico Gemelli, Università Cattolica, Rome, Italy

**Keywords:** Cerebrovascular malformations, Secondary headache disorders, Imaging, Angiography

## Abstract

**Background:**

Dural arteriovenous fistulas are intracranial vascular malformations, fed by dural arteries and draining venous sinuses or meningeal veins. Clinical course varies widely and ranges from benign with spontaneous remission to fatal, due to cerebral hemorrhage. In a 10-year single institution experience, clinical presentation of dural arteriovenous fistulas, and in particular headache and angiographic features, as well as long-term outcome were analyzed.

**Methods:**

Data of 42 intracranial dural arteriovenous fistulas of 40 patients concerning demographic characteristics, medical history and risk factors, clinical presentation and headache features, location and neuroimaging findings, as well as treatment and outcome, were collected. Furthermore, we used the modified-Rankin Scale to assess the long-term outcome, by telephone contact with patients and/or their relatives.

**Results:**

Patients aged between 25 and 89 years (mean age 55.8 ± 15.5). According to different clinical presentation and evolution, related to their unique drainage pattern into the cavernous sinus, we examined the carotid-cavernous fistulas separately from other dural arteriovenous fistulas. Interestingly, we found that the *migraine-like* headache was the major onset symptom of dural arteriovenous fistulas different from carotid-cavernous fistulas (*p* = 0.036). On the other hand, *non-migraine-like* headache was a typical characteristic of carotid-cavernous fistulas (*p* = 0.003). Moreover, ocular symptoms were more frequently observed in carotid-cavernous fistulas (92.9% *p <* 0.001). Seventy percent of patients did not report any impact on quality of life (mRS 0 or 1) at follow-up.

**Conclusions:**

These findings suggest a link between the site of lesion and clinical features of the headache, a symptom that usually leads to hospitalization. In particular, ocular symptoms accompanying *non-migraine-like* headache should be promptly recognized and raise the suspicion of a carotid-cavernous fistula, while *migraine-like headache* may suggests other dural arteriovenous fistulas. This study provides new significant insights on headache and its characteristics as a presentation symptom in dural arteriovenous fistulas.

## Background

Dural arteriovenous fistulas (DAVFs) are peculiar intracranial vascular malformations, fed by dural arteries and draining venous sinuses or meningeal veins. They constitute up to10–15% of cerebral vascular malformations [1].

The DAVFs etiology remains largely uncertain. Regardless of the causes, DAVFs are considered acquired rather than congenital lesions, assuming that the intracranial venous sinus hypertension leads to the development of fistulous connections between the arterial and the venous side of the dural wall [2–4]. Intracranial venous sinus hypertension is mainly caused by head trauma, infections, tumors, previous craniotomy or dural venous sinus thrombosis [2–4].

The clinical course of DAVFs varies widely and ranges from benign with spontaneous remission to fatal due to cerebral hemorrhage [5].

Furthermore, the correlation between the venous drainage pattern, evaluated by conventional angiography, and the various clinical signs and symptoms defines diagnosis and classification of DAVFs. In fact, high-grade DAVFs and retrograde leptomeningeal venous drainage seem to be related to a more aggressive neurological presentation [6–9].

DAVFs treatment includes different options: conservative, endovascular (trans-arterial or trans-venous embolization), surgical or stereotactic radiosurgery approaches. Venous drainage patterns and the risk of an aggressive clinical presentation, as well as presenting symptoms, and their impact on quality of life, determine the choice of the most appropriate treatment [10–13].

Low occurrence of DAVFs justifies the limited availability of data about clinical features of these lesions. Moreover, although previous studies have reported headache as frequently associated with DAVFs, they have never been investigated the features of headache in DAVFs in relationship with neuroimaging.

## Methods

The aim of this study is to show a 10-year single institution experience with diagnosed and/or treated DAVFs, analyzing their clinical presentation with a particular attention on headache characteristics and angiographic features, as well as their long-term outcome. Our Institutional Review Board approved this observational study. For each patient with a diagnosis of DAVF, we collected data about demographic characteristics, medical history and risk factors, clinical presentation, location as well as treatment and outcome. In particular, as for as the headache characteristics (pre-existing in medical history and/or new-onset in clinical presentation), data about period of onset, duration, location, quality, intensity, aggravation by routine physical activity and associated symptoms were collected. Neuroimaging features were classified according to Barrow, Borden and Cognard [7–9]. As shown in Table 1, the Barrow’s classification is the one usually assumed for carotid-cavernous fistulas (CCFs), meanwhile the Cognard’s classification is the one most widely used for other DAVFs than CCFs.

**Table 1:** Barrow’s and Cognard’s classifications of DAVFs

Barrow (for CCFs)		Cognard (for other DAVFs than CCFs)	
A	Direct shunting of blood flow from the ICA into the cavernous sinus.	I	Normal anterograde flow into dural sinus
		IIa	Retrograde flow into dural venous sinus(es)
B	Shunts to the cavernous sinus from branches of the ICA	IIb	Anterograde flow into dural venous sinus and retrograde filling of cortical vein(s)
		IIa + b	Retrograde drainage into sinus(es) and cortical vein(s)
C	Shunts to the cavernous sinus from branches of the ECA	III	Direct drainage into cortical veins without venous ectasia
		IV	Direct drainage into cortical veins with venous ectasia (> 5 mm and 3 times larger than diameter of draining vein)
D	Shunts from both the ICA and ECA simultaneously	V	Drainage to spinal perimedullary veins

Abbreviations: *ICA* Internal Carotid Artery, *ECA* External Carotid Artery. Adapted from References 7 and 9

Patients’ medical history was assessed with regard to alcohol and smoke habit; previous pregnancies and/or miscarriages; menopause status; family history (such as vascular and neurological diseases); personal history of epilepsy, headache, head trauma or neurosurgery; vascular risk factors (such as hypertension, atrial fibrillation, myocardial infarction, chronic ischemic heart disease or other cardiopathies, vasculitis, antiphospholipid syndrome, thrombophilia, hereditary haemorrhagic telangiectasia, transient ischemic attack (TIA), stroke, cerebral hemorrhage); previous or concurrent gastroenteric/lung/kidney/eye diseases; endocrinopathies; psychiatric diseases and other neurological diseases.

Furthermore, to assess the long-term outcome, we used the modified-Rankin Scale (mRS) [14] calculated by a telephone contact with patients and their relatives (in January 2019). MRS is a 7-level ordered categorical scale capturing levels of patient functional independence following a cerebrovascular accident, with scores ranging from 0 (fully independent) to 6 (dead).

### Statistical analysis

Statistical analyses were performed using R software version 3.1 [15]. Continuous variables were described by means and standard deviations, while categorical ones were reported as count and percentages. Fisher exact test was performed to test significance of associations between categorical variables. Significance level of 5% was assumed for all the analyses.

## Results

**Fig. 1:**
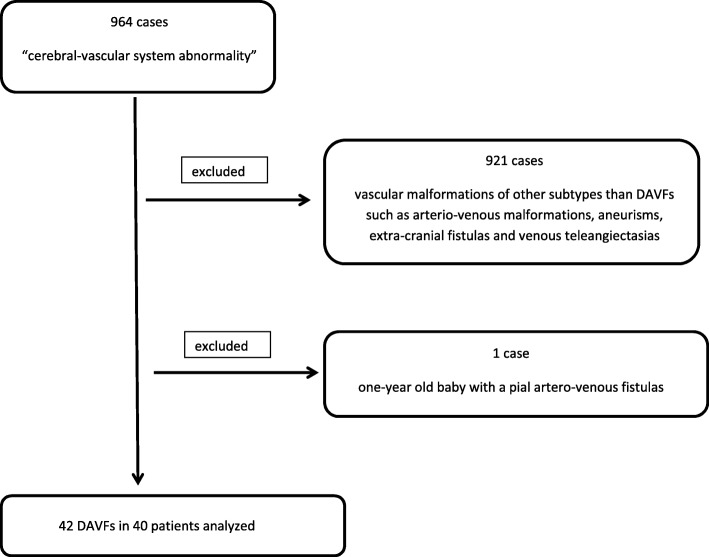
Flow-chart of the selection process

Looking for patients discharged from our Hospital in a 10-year period (from 1st January 2008 to 31th December 2018) with the diagnosis of a “cerebral-vascular system abnormality”, we found 964 cases: 921 were excluded because they were vascular malformations of other subtypes than DAVFs such as arterio-venous malformations, aneurisms, extra-cranial fistulas and venous teleangiectasias. Furthermore, one-year old baby with a pial artero-venous fistulas was also excluded (see Fig. 1).

Finally, we found 42 DAVFs in 40 patients aged between 25 and 89 years (mean age 55.8 ± 15.5) at the time of the diagnosis. Twenty-one (52.5%) patients were women. According to the well-known different clinical presentation and evolution related to their unique drainage pattern into the cavernous sinus [16, 17] we examined CCFs separately from other DAVFs. No significant differences were found between the age and sex subgroups in relation to the type of DAVFs.

### Location

**Fig. 2:**
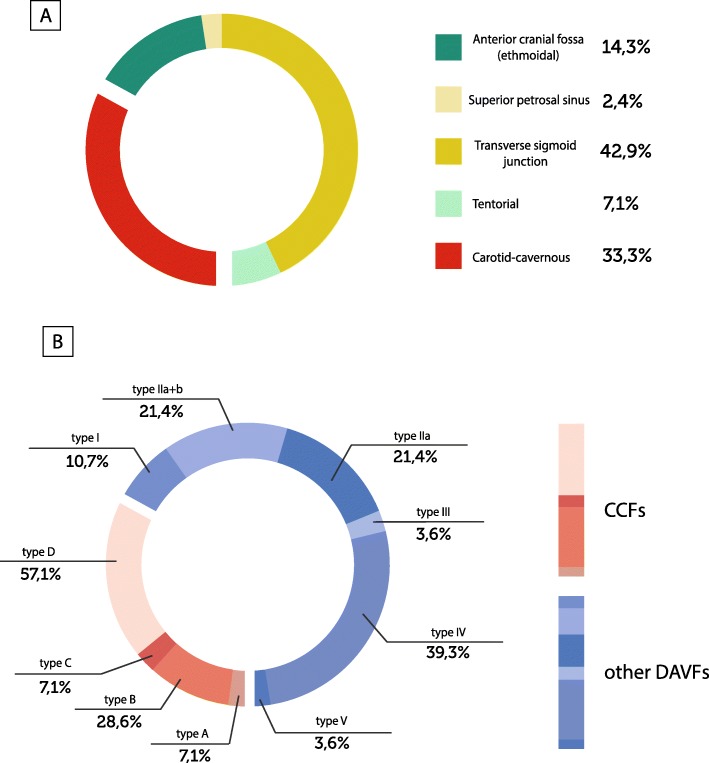
Subtypes of DAVFs according to angiographic features, Barrow’s and Cognard’s classifications. *A:* Location of DAVFs according to angiographic features with related percentages of presentations of our series. None superior sagittal sinus DAVF detected. *B:* Type of CCFs, according to Barrow’s classification (types A, B, C, D) and type of other DAVFs than CCFs, according to Cognard’s classification (types I, IIa, IIa + b, III, IV, V) and related percentages of presentations of our series. None type IIb detected. Abbreviations: CCFs = carotid-cavernous fistulas; DAVFs = dural arteriovenous fistulas

At the admission, a cranial CT and/or MRI scan were done for a first line diagnosis; then each patient underwent, after a written consent, a complete cerebral angiography. Fig. 2 (A and B) shows all 42 DAVFs divided according to the angiographic features [17]. None superior sagittal sinus DAVF was detected. Two patients had a double-lesion: 1 of them had 1 tentorial DAVF and 1 CCF, meanwhile the other one had 1 left and 1 right CCF.

### Clinical presentation

Table 2 summarizes the presentation symptoms of the DAVFs, while for neurological examination at hospital arrival see Additional File 1. The total number of symptoms/signs exceeds 40 because many patients had multiple complains. Symptomatic patients came to the hospital due to recent onset of symptoms. Among them, the most common complained symptom was headache (45.2%), without a significant difference among the DAVFs subtype. According to clinical features, this presentation symptom was defined as follows: *migraine-like* headache if the clinical features fulfilled those described by the International Headache Society (IHS) classification criteria [18] for migraine, or as *non-migraine-like* headache. For this latter, clinical features were referred as: orbital/peri-orbital/supra-orbital location, unilateral (right or left) pain, variability in intensity ranging from mild to severe, variability in quality because referred as constrictive/oppressive/pulsating/stabbing, neither worsening by physical effort nor accompanying by other symptoms such as phono/photophobia. Interestingly, we found that the *migraine-like* headache seems to be a typical characteristic of DAVFs different from CCFs (*p* = 0.036). On the other hand, *non-migraine-like* headache was a typical characteristic of CCFs (*p* = 0.003). In all *non-migraine-like* cases, the side of headache was ipsilateral to the side of the fistula, while in *migraine-like* cases there was no significant side correlation. Moreover, ocular symptoms were more frequently observed in CCFs (92.9% *p <* 0.001).

**Table 2:** Symptoms in relation to the type of DAVFs

Clinical presentation	All N (%)	CCFs N (%)	Other DAVFs N (%)	*p value*
N	40	14 (35.0)	26 (65.0)	
Ocular complaints	15 (37.5)	13 (92.9)	2 (7.7)	< 0.001
Diplopia	11 (27.5)	11 (78.6)	0	< 0.001
Ptosis	2 (5.0)	2 (14.3)	0	*ns*
Exophtalmos	5 (12.5)	5 (35.7)	0	0.003
Hyperemia / conjunctival chemosis	9 (22.5)	9 (64.3)	0	< 0.001
Campimetric deficit	3 (7.5)	1 (7.1)	2 (7.1)	*ns*
Half-face hypoesthesia	1 (2.5)	1 (7.1)	0	*ns*
Headache	18 (45.0)	7 (50.0)	11 (42.3)	*ns*
Non-Migraine-like headache	6 (15.0)	6 (42.9)	0	< 0.001
Migraine-like headache	12 (30.0)	1 (7.1)	11 (42.3)	0.03
Cerebellar/hearing/vestibular dysfunction	12 (30.0)	1 (7.1)	9 (34.6)	*ns*
Nausea / vomiting	1 (2.5)	0	1 (3.8)	*ns*
Postural instability	2 (5.0)	0	2 (7.7)	*ns*
Tinnitus / hearing loss	7 (17.5)	1 (7.1)	6 (23.1)	*ns*
Other cranial nerves complaints	2 (5.0)	2 (14.3)	0	*ns*
Laterocervical / retroauricolar pain	3 (7.5)	1 (7.1)	2 (7.7)	*ns*
Seizures	2 (5.0)	1 (7.1)	1 (3.8)	*ns*
Aphasia	1 (2.5)	0	1 (3.8)	*ns*
Limbs weakness	1 (2.5)	0	1 (3.8)	*ns*
Hemisoma/limbs paresthesia/hypoesthesia	3 (7.5)	1 (7.1)	1 (3.8)	*ns*
Sphincter disorders	1 (2.5)	0	1 (3.8)	*ns*
Confusional state	2 (5.0)	0	2 (7.7)	*ns*
Syncope	1 (2.5)	0	1 (3.8)	*ns*
Asymptomatic	5 (12.5)	0	4 (15.4)	*ns*

Abbreviations: *CCFs* carotid-cavernous fistulas, *DAVFs* dural arteriovenous fistulas, ns not significant

Finally, 12.5% of the patients performed a neuroimaging examination even if asymptomatic (e.g.: family member suffering from intracerebral vascular malformation or other brain pathology, exclusion of contraindications to particular sport activities).

### Risk factors and medical history

The most frequent disease in personal history was hypertension (45.0%), followed by headache (30.0%) and dyslipidemia (27.5%). In particular, in the headache cohort, 2 (16.7%) patients had history of migraine with aura, whereas the others complained other type of primary headache, according to IHS criteria [18] (see Additional File 2 for details). There were no significant differences between the explored subgroups in terms of personal history and risk factors, except for the smoking habits. In particular, given that the headache reported at the admission was referred as extremely different in characteristics from that referred in history, we also evaluated if being a subject with headache lead to having headache as a clinical presentation symptom of the DAVF: combining data about headache, both as a presentation symptom and as symptom present in personal history, we don’t find a significant association with any of the subtypes of DAVFs. As shown in Table 3, three (7.5%) patients have had a previous ischemic stroke/TIA, 5 (11.9%) patients showed some kind of peripheral thrombosis (inferior and/or superior limbs, retinal), 1 (4.2%) patient had a recognized antiphospholipid syndrome and 1 (4.3%) had a hereditary thrombotic disease. Notably, only 4 (10.0%) patients had a history of head trauma, 2 of which occurred just a few months before the diagnosis of DAVF. The presumed etiology of our case series, was idiopathic in 88.1% of cases; in only 4.8% and 7.1% was found respectively a head traumatic cause or cerebral venous thrombosis, in close temporal relation with the diagnosis of DAVFs.

**Table 3:** Patients’ history and risk factors. (see page 7)

	All N (%)	CCFs N (%)	Other DAVFs N (%)	*p value*
N	40	14 (35.0)	26 (65.0)	
Alcohol use				*ns*
*No*	15 (39.5)	5 (35.7)	10 (41.7)	
*Yes*	0 (0.0)	0	0	
*Sporadic*	23 (60.5)	9 (64.3)	14 (58.3)	
*Missing*	2	0	2	
Smoking				
*No*	26 (68.4)	13 (92.9)	13 (54.2)	0.014
*Previous use*	9 (23.7)	0	9 (37.5)	*ns*
*Yes*	3 (7.9)	1 (7.1)	2 (8.3)	*ns*
*Missing*	2	0	2	
Epilepsy				*ns*
*Yes*	3 (8.1)	2 (15.4)	1 (4.2)	
*No*	34 (91.9)	11 (84.6)	23 (95.8)	
*Missing*	3	1	2	
Headache				*ns*
*Yes*	12 (23.1)	3 (23.1)	9 (36.0)	
*No*	26 (76.9)	10 (76.9)	16 (64.0)	
*Missing*	2	1	1	
Head trauma				*ns*
*Yes*	4 (10.0)	1 (7.1)	3 (11.5)	
*No*	36 (90.0)	13 (92.9)	23 (88.5)	
*Missing*	0	0	0	
Stroke/TIA				*ns*
*Yes*	3 (7.5)	2 (14.3)	1 (3.8)	
*No*	37 (92.5)	12 (85.7)	25 (96.2)	
*Missing*	0	0	0	
Cerebral hemorrhage				*ns*
*Yes*	1 (2.5)	0 (0.0)	1 (3.8)	
*No*	39 (97.5)	14 (100.0)	25 (96.2)	
*Missing*	0	0	0	
Thrombosis				*ns*
*Yes*	5 (11.9)	2 (14.3)	3 (11.5)	
*No*	35 (88.1)	12 (85.7)	23 (88.5)	
*Missing*	0	0	0	
Vascular risk factors				
*Antiphospholipid syndrome*				*ns*
*Yes*	1 (4.2)	0 (0.0)	1 (6.7)	
*No*	23 (95.8)	9 (100.0)	14 (93.3)	
*Missing*	16	5	11	
*Trombophilia*				*ns*
*Yes*	1 (4.3)	0 (0.0)	1 (7.1)	
*No*	22 (95.7)	9 (100.0)	13 (92.9)	
*Missing*	17	5	12	

Abbreviations: *CCFs* carotid-cavernous fistulas; *DAVFs* dural arteriovenous fistulas; *TIA* transient ischemic attack; *ns* not significant

### Angiographic features

Angiographic features are shown in Table 4. In our series, the majority of venous drainage was in dural sinus (54.8%), while it was both in dural sinus and in cortical veins in 4 (9.5%) patients and in perimedullary veins in 1 (2.4%) patient. A concomitant venous ectasia was present in 55.3% of cases. In 54.2% of our cases, the anterior-inferior dural sinus was involved, mainly in the case of CCFs (*p* < 0.001). Furthermore, we found both anterograde and retrograde drainage in 7 (16.7%) patients. The diagnosis of DAVF was found in only 3 (7.1%) patients with a concomitant cerebral venous thrombosis. None of these had seizures as a presentation symptom or a history of thrombosis. Only 1 patient with concomitant cerebral venous thrombosis had headache as a presentation symptom. The angiographic examination has allowed the grading of DAVFs according to Barrow’s classification (for CCFs) and Cognard classification (for other DAVFs different from CCFs), as shown in Fig. 1 [7–9]. The majority (57.1%) of CCFs were classified as type D, while the majority (39.3%) of the other DAVFs were classified as type IV. In our series, there were no IIb type of DAVFs. Sixteen (40%) patients came to the hospital with aggressive symptoms such as hemorrhage, seizures, focal neurological deficits, cranial nerve palsies, myelopathy or intracranial hypertension signs. Of these, 2 had a generalized seizure, 1 of which as the presentation symptom of a brain parenchymal hemorrhage.

**Table 4:** Angiographic features

Angiographic features	All N (%)	CCFs N (%)	Other DAVFs N (%)	*p value*
N	42	14 (33.3)	28 (66.7)	
DAVF side				
Left	20 (47.6)	5 (35.7)	15 (53.6)	*ns*
Right	18 (42.9)	7 (50.0)	11 (39.3)	*ns*
Midline	4 (9.5)	2 (14.3)	2 (7.1)	*ns*
Venous drainage				
Dural sinus	23 (54.8)	10 (71.4)	13 (46.4)	*ns*
Cortical veins	14 (33.3)	2 (14.3)	12 (42.9)	*ns*
Both	4 (9.5)	2 (14.3)	2 (7.1)	*ns*
Perimedullary veins	1 (2.4)	0	1 (3.6)	*ns*
Dural sinus specification				
Anterior-inferior	13 (54.2)	11 (100.0)	2 (15.4)	< 0.001
Posterior-superior	11 (45.8)	0	11 (84.6)	*ns*
Missing	18	3	15	
Drainage				
Anterograde	13 (36.1)	2 (15.4)	11 (47.8)	*ns*
Retrograde	17 (47.2)	8 (61.5)	9 (39.1)	*ns*
Both	6 (16.7)	3 (23.1)	3 (13.1)	*ns*
Missing	6	1	5	
High flow	2 (12.5)	1 (8.3)	1 (25.0)	*ns*
Concomitant venous ectasia	21 (55.3)	7 (53.8)	14 (56.0)	*ns*
Concomitant sinus thrombosis	3 (7.1)	1 (7.1)	2 (7.1)	*ns*

Abbreviations: *CCFs* carotid-cavernous fistulas, *DAVFs* dural arteriovenous fistulas, *ns* not significant

### Treatment

A conservative approach was chosen in 6 (14.3%) out of the 42 DAVFs, 24 patients (66.6%) were treated by trans-arterial approach, while 12 (33.3%) were treated by trans-venous approach. Based upon the post-embolization angiographic assessment, the treatment resulted in a complete occlusion of the DAVF in 66.7% of cases (see Additional File 3). Major peri-procedural complications did not occur.

### Long-term outcome

**Fig. 3:**
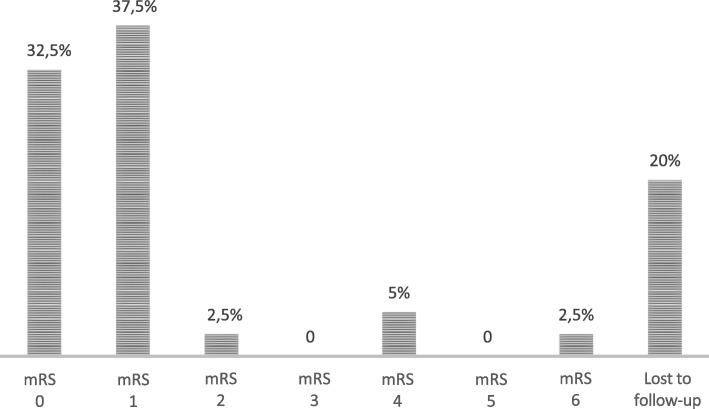
Outcome (mRS) of patients at follow-up. Abbreviations: mRS = modified Rankin Scale

In the follow-up some of the patients reported more than one hospitalization due to DAVF, the number of which ranges from 1 to 5. Follow-up, from the first hospitalization to the 2019 telephone contact, ranged from 8 to 178 months (mean 74 ± 43 months). As shown in Fig. 3, among 40 patients, 8 (20%) were lost to follow-up; 70% did not report any consequence on quality of life (asymptomatic or without significant disability, mRS 0 or 1). One patient died due to intracranial hemorrhage 2 years after the diagnosis of DAVFs.

## Discussion

Data regarding location, etiology, clinical presentation, prognosis and treatment of DAVFs are available from multiple case series and review published during the last decades [16, 17, 19–22]. Despite this, due to low presentation rate of this kind of lesions, the diagnosis of DAVFs is not as easy as it may appear. In light of this, we reported all the retrieved characteristics of our patients for updating purposes.

In accordance to literature findings [17], the transverse-sigmoid junction is the most common site of localization of our series of DAVFs, followed by CCFs. On the other hand, we did not observe cases of superior sagittal sinus DAVF, while they are reported in literature to be approximately 8–13% of all intracranial fistulae [7, 17]. According to literature showing that multiple shunts can occur, in our series we found 2 double DAVFs [23].

Symptoms depend on DAVF location and the pattern of the venous drainage. In particular, CCFs often present with ocular symptoms, due to the reversal of flow in the ophthalmic veins related to sinus increased pressure [20]. On the other hand, tinnitus, headache and progressive neurologic deficits are more typical of other types of DAVFs [17]. In our series, the presentation symptoms of all kind of DAVFs is headache. However, a critical difference was observed between the two groups of DAVFs. In fact, while CCFs showed a *non-migraine like headache* frequently associated with an ocular complain, other DAVFs had a *migraine like headache.*

Indeed, headache is known as a common onset symptom of DAVF, usually described as localized to the same site of the lesion, becoming generalized as a result of the dural stretching [17]. The International Headache Society (IHS) has drawn up the classification criteria for the headache associated with DAVF [18] taking into account: (i) temporal relation to other symptoms/signs of DAVF; (ii) parallel prognosis between DAVF and headache; (iii) association with other signs/symptoms (such as tinnitus, ophtalmoplegia) and its clinical course (progressive, worse in the morning or due to a Valsalva’s maneuver); (iv) same localization of headache and site of DAVF. Nevertheless, none of the previous studies on DAVFs have classified headaches according to the IHS classification criteria [18]. Thus, a description of headache characteristics according to IHS classification criteria was an unmet need that has been addressed in our study.

Our findings do not show a significant correlation between the headache as a clinical onset and the headache already present in personal history, while suggesting a link between the site of the lesion and the clinical features of the headache, symptom that led to hospitalization. This does not unequivocally exclude that the DAVF had always been there, but since the characteristics of the headache have suddenly changed leading the patient to go to the emergency room, the influence of the previous headache appears unlikely to us.

We can speculate that our observation can be explained by the current theories about migraine pathophysiology. In fact, although the exact pathophysiological mechanism underlying migraine is still not completely understood, it is known that trigeminal activation is accompanied by the release of vasoactive neuropeptides producing a sterile local inflammatory response, called *neurogenic inflammation*, within the algo-sensitive intracranial tissues such as the meninges [24, 25]. In particular, the release of neuropeptides (including calcitonine gene related peptide, CGRP) by trigeminal sensitive nerve endings leads to marked arteriolar vasodilation and plasma extravasation with inflammatory edema, mostly nearby small arterioles (middle meningeal artery and related arterioles) and post-capillary venules [25–28]. The vascular effects of CGRP appear more pronounced in the microvasculature, such as muscular tunic of smaller caliber arteries at the meningeal level [25–28].

We could hypothesize that the increase in vascular pulsatility in these arteriolar and venous territories might lead to an increased activity of the perivascular sensory terminations present at the DAVFs level. These events might trigger the release of neuropeptides from the trigeminal sensitive nerve peripheral endings at the meningeal level, where they can evoke components of the *neurogenic inflammation* underlying *migraine-like headache* in DAVFs other than CCF. In fact, the CCFs involve the internal or external carotid artery (ICA, ECA) and the cavernous sinus that have greater caliber than the meningeal arterioles. On the other hand, ocular symptoms accompanying *non-migraine-like* headache should be promptly recognized and raise suspicion of a CCF in these patients.

Regarding previously described risk factors [20, 22, 29–31], only in a few patients we found a positive history of head trauma or cerebral venous thrombosis, in close temporal relation with the diagnosis of DAVFs.

Potential major morbidity associated with the endovascular treatment it is estimated to be 3% or less [32]. Endovascular approach in our case series was successfully utilized in the large majority of patients. Peri-procedural major complications were not observed.

Follow-up outcome assessment revealed that a few patients had significant disability related to DAVFs and need for assistance with an mRS of 4 at follow-up.

The major strength of our study is the use of IHS classification that adds valuable information on headache features in these peculiar patients. Moreover, the long-term follow-up is another strength of our study. There are some limitations in our study. Indeed, we used clinical-based samples rather than population-based ones. Thus, a selection bias cannot be excluded since our cohort is represented by patients admitted to a single institution. However, in relation to the collection of medical history and clinical data, conducted by two different researchers, information and recall bias cannot be excluded. At last, the rare occurrence of DAVFs and the consequent small sample size collected could lead to inconclusive results, especially regarding possible correlation between angiographic features and headache type. A larger sample size could lead to a more accurate statistical analysis. Certainly, prospective studies, including a more accurate assessment of the progress of headache following DAVF treatment, are necessary.

## Conclusions

Although our study is in line with the previous clinical studies on DAVFs, it provides new significant insights on clinical presentation regarding the characteristics of headache. Hopefully, future multicenter studies, with larger sample size, will help to clarify and specify the correlation between imaging features of lesions and characteristics of headaches in DAVFs. Indeed, there are several points that remain to be better evaluated, such as the evolution of the headache following the DAVF treatment. Surely, a study with a full prospective setting could clarify these aspects.

## Supplementary information

**Additional file 1.** Neurological examination at hospital arrival.

**Additional file 2.** Details of the 12 patients with history of headache in anamnesis.

**Additional file 3.** Treatment approaches.

## Data Availability

The datasets used and/or analysed during the current study are available from the corresponding author on reasonable request.

## Abbreviations

CCFs: Carotid-cavernous fistulas
CGRP: Calcitonine gene related peptide
DAVFs: Dural arteriovenous fistulas
ECA: External carotid artery
ICA: Internal carotid artery
IHS: International Headache Society
mRS: Modified-Rankin Scale
TIA: Transient ischemic attack
