# Supplementary material for: Dural arteriovenous fistulas and headache features: an observational study
Source: J Headache Pain. 2020 Jan 16;21(1):6. doi: 10.1186/s10194-020-1073-1 (PMC6966899; doi:10.1186/s10194-020-1073-1)
Supplement: Supplementary file 1 — Additional file 1. Neurological examination at hospital arrival. [file 10194_2020_1073_MOESM1_ESM.doc]

**Additional File 1. Neurological examination at hospital arrival.**

| **Neurological examination** | **All**  **N (%)** | **CCFs**  **N (%)** | **Other DAVFs**  **N (%)** | ***p value*** |
| --- | --- | --- | --- | --- |
| **N** | 40 | 14 (35.0) | 26 (65.0) | *ns* |
| Unilateral IIIrd cranial nerve palsy | 1 (2.5) | 1 (7.1) | 0 | *ns* |
| Bilateral IIIrd cranial nerve palsy | 1 (2.5) | 1 (7.1) | 0 | *ns* |
| Unilateral VIth cranial nerve palsy | 8 (20.0) | 8 (57.1) | 0 | <0.001 |
| Campimetric deficit | 1 (2.5) | 0 | 1 (3.8) | *ns* |
| Papilledema | 1 (2.5) | 0 | 1 (3.8) | *ns* |
| Bilateral ophthalmoplegia | 2 (5.0) | 2 (14.3) | 0 | *ns* |
| Unilateral VIIth cranial nerve palsy | 3 (7.5) | 2 (14.3) | 1 (3.8) | *ns* |
| Emisoma/limbs sensitive disturbances | 4 (10.0) | 1 (7.1) | 3 (11.5) | *ns* |
| Face sensitive disturbances | 1 (2.5) | 1 (7.1) | 0 | *ns* |
| Limbs dysmetria | 1 (2.5) | 1 (7.1) | 0 | *ns* |
| Nystagmus | 4 (10.0) | 1 (7.1) | 3 (11.5) | *ns* |
| Trunk/limbs/walk ataxia | 2 (5.0) | 0 | 2 (7.7) | *ns* |
| Hearing loss | 1 (2.5) | 0 | 1 (3.8) | *ns* |
| Retro-auricolar bruit | 5 (12.5) | 1 (7.1) | 4 (15.4) | *ns* |
| Tetraparesis | 1 (2.5) | 0 | 1 (3.8) | *ns* |
| Sphincter disorders | 1 (2.5) | 0 | 1 (3.8) | *ns* |
| Anisocoria | 2 (5.0) | 1 (7.1) | 1 (3.8) | *ns* |
| Consciousness disturbance | 1 (2.5) | 0 | 1 (3.8) | *ns* |

Abbreviations: CCFs = carotid-cavernous fistulas; DAVFs = dural arteriovenous fistulas; *ns* = not significant.
