# Supplementary material for: Dural arteriovenous fistulas and headache features: an observational study
Source: J Headache Pain. 2020 Jan 16;21(1):6. doi: 10.1186/s10194-020-1073-1 (PMC6966899; doi:10.1186/s10194-020-1073-1)
Supplement: Supplementary file 2 — Additional file 2. Details of the 12 patients with history of headache in anamnesis. [file 10194_2020_1073_MOESM2_ESM.doc]

**Additional file 2. Details of the 12 patients with history of headache in anamnesis.**

1. Male, 47yrs, episodic tension-type headache since adolescence, no headache at admission;

2. Male, 44yrs, episodic tension-type headache since adolescence, recent onset of chronic migraine-like headache at admission;

3. Male, 66yrs, episodic migraine from adolescence to 55yrs, then remission of headache, recent onset of chronic migraine-like headache at admission;

4. Female, 39yrs, episodic tension-type headache since 30yrs, no headache at admission;

5. Female, 51yrs, episodic migraine since adolescence, no headache at admission;

6. Female, 38 yrs, menstrual migraine since adolescence, no headache at admission;

7. Female, 54yrs, episodic tension-type headache since adolescence, recent onset of chronic migraine-like headache at admission;

8. Female, 54yrs, episodic migraine with aura since adolescence, recent onset of chronic migraine-like headache without aura, associated to tinnitus and hearing loss at admission.

9. Female, 88yrs, episodic tension-type headache from adolescence to 60yrs, recent onset of chronic non-migraine-like headache at admission;

10. Female, 66yrs, menstrual migraine since adolescence to 48yrs, then remission of headache, recent onset of chronic migraine-like headache at admission;

11. Male, 55yrs, episodic tension-type headache since 25yrs, recent onset of chronic migraine-like headache at admission;

12. Female, 46yrs, episodic migraine with aura since 22yrs, recent onset of chronic migraine-like headache without aura at admission.
