# Supplementary material for: Dural arteriovenous fistulas and headache features: an observational study
Source: J Headache Pain. 2020 Jan 16;21(1):6. doi: 10.1186/s10194-020-1073-1 (PMC6966899; doi:10.1186/s10194-020-1073-1)
Supplement: Supplementary file 3 — Additional file 3. Treatment approaches. [file 10194_2020_1073_MOESM3_ESM.doc]

***Additional File 3. Treatment approaches.***

| **Treatment approaches** | **All**  N (%) | **CCFs**  **N (%)** | **Other DAVFs**  **N (%)** | ***p value*** |
| --- | --- | --- | --- | --- |
| **N** | 42 | 14 (33.3) | 28 (66.7) |  |
| **Endovascular treatment** | 36 (83.3) | 11 (78.6) | 25 (89.3) | *ns* |
| Arterial | 24 (66.6) | 1 (7.1) | 23 (82.1) | *<0.001* |
| Venous | 12 (33.3) | 10 (71.4) | 2 (7.1) | *ns* |
| **Untreated** | 6 (14.3) | 3 (21.4) | 3 (10.7) | *ns* |
| **Complete occlusion** | 24 (66.7) | 7 (63.6) | 17 (68.0) | *ns* |

Abbreviations: CCFs = carotid-cavernous fistulas; DAVFs = dural arteriovenous fistulas; *ns* = not significant.
